# Absolute cardiovascular risk assessment using ‘real world’ clinic blood pressures compared to standardized unobserved and ambulatory methods: an observational study

**DOI:** 10.1038/s41440-024-01841-1

**Published:** 2024-08-16

**Authors:** Niamh Chapman, Senali Jayasinghe, Myles N. Moore, Dean S. Picone, Martin G. Schultz, Matthew D. Jose, Roland W. McCallum, Matthew K. Armstrong, Xiaoqing Peng, Thomas H. Marwick, Philip Roberts-Thomson, Nathan B. Dwyer, J. Andrew Black, Mark R. Nelson, James E. Sharman

**Affiliations:** 1 Menzies Institute for Medical Research, University of Tasmania, Hobart, TAS Australia; 2 School of Health Sciences, Faculty of Medicine and Health, University of Sydney, Camperdown, NSW Australia; 3 Renal Unit, Royal Hobart Hospital, Hobart, TAS Australia; 4 School of Medicine, University of Tasmania, Hobart, TAS Australia; 5 Department Diabetes and Endocrine Services, Royal Hobart Hospital, Hobart, TAS Australia; 6 Department of Health and Human Physiology, University of Iowa, Iowa, IA USA; 7 Department of Obstetrics and Gynaecology, The First Affiliated Hospital of Anhui Medical University, Anhui, China; 8 Baker Heart and Diabetes Institute, Melbourne, VIC Australia; 9 Royal Hobart Hospital, Hobart, TAS Australia

**Keywords:** Mass screening, Cholesterol, Telemedicine

## Abstract

Clinic blood pressure (BP) is recommended for absolute cardiovascular disease (CVD) risk assessment. However, in ‘real-world’ settings, clinic BP measurement is unstandardised and less reliable compared to more rigorous methods but the impact for absolute CVD risk assessment is unknown. This study aimed to determine the difference in absolute CVD risk assessment using real-world clinic BP compared to standardised BP methods. Participants were patients (*n* = 226, 59 ± 15 years; 58% female) with hypertension referred to a BP clinic for assessment. ‘Real-world’ clinic BP was provided by the referring doctor. All participants had unobserved automated office BP (AOBP) and 24-h ambulatory BP monitoring (ABPM) measured at the clinic. Absolute CVD risk was calculated (Framingham) using systolic BP from the referring doctor (clinic BP), AOBP and ABPM, with agreement assessed by Kappa statistic. Clinic systolic BP was 18 mmHg than AOBP and daytime ABPM and 22 mmHg higher than 24-h ABPM (*p* < 0.001). Subsequently, absolute CVD risk scores using clinic BP were higher compared to AOBP, daytime ABPM and 24-h ABPM (10.4 ± 8.1%, 7.8 ± 6.4%, 7.8 ± 6.3%, and 7.3 ± 6.1%, respectively, *P* < 0.001). As a result, more participants were classified as high CVD risk using clinic BP (*n* = 89, 40%) compared with AOBP (n = 44, 20%) daytime ABPM (*n* = 38, 17%) and 24-h ABPM (*n* = 38, 17%) (*p* < 0.001) with weak agreement in risk classification (κ = 0.57[0.45–0.69], κ = 0.52[0.41–0.64] and κ = 0.55[0.43–0.66], respectively). Real-world clinic BP was higher and classified twice as many participants at high CVD risk compared to AOBP or ABPM. Given the challenges to high-quality BP measurement in clinic, more rigorous BP measurement methods are needed for absolute CVD risk assessment.

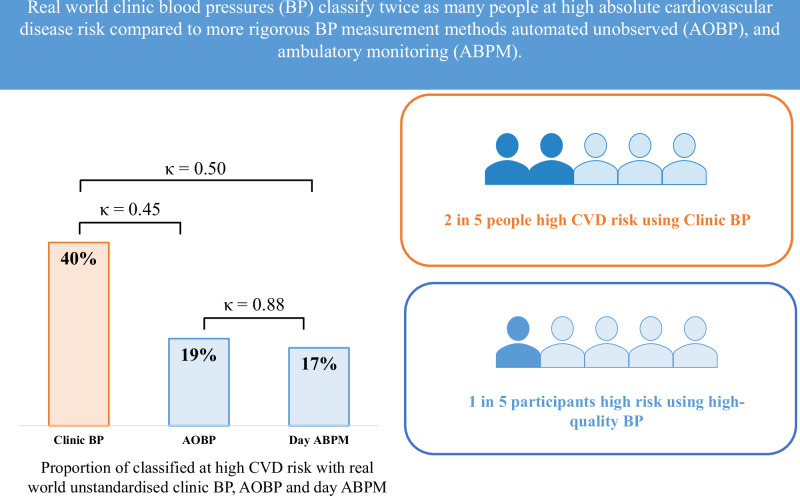

## Introduction

Guidelines for the primary prevention of cardiovascular disease (CVD) recommend absolute risk assessment to identify patients at high risk of CVD based on multiple risk factors [1]. Blood pressure (BP) is the single most important modifiable risk factor for the prevention of CVD and an integral component of absolute CVD risk assessment [2]. Several absolute CVD risk assessment equations recommend the use of a systolic BP value recorded in the clinic environment with the patient observed by a nurse or doctor [3–6]. However, there are many sources of inaccuracy and challenges associated with BP measurement in busy clinical practice, which often leads to different BP readings compared with out-of-clinic BP methods [7].

More rigorous BP measurement methods have been recommended to address the barriers to high-quality clinic BP measurement, including unobserved automated office BP (AOBP) and the gold-standard 24-h ambulatory BP monitoring (ABPM) [8, 9]. However, these higher quality BP measurement methods are not recommended for use in absolute CVD risk assessment because the risk algorithms were originally generated using a high-quality, standardized, research-grade protocol to represent ‘clinic BP’ [3, 6]. This is not how clinic BP is measured in real world clinical practice, where BP values are more likely to overestimate the true underlying BP [10, 11]. Therefore, the use of real-world clinic BP for absolute CVD risk assessment may lead to overestimation of risk. To our knowledge the effect of using out-of-clinic BP measurement and unobserved AOBP for absolute CVD risk calculation has not been investigated previously. The aim of this study was to compare the use of real-world clinic BP to unobserved AOBP and the gold-standard ABPM for absolute CVD risk assessment.

## Methods

### Study protocol

For this cross-sectional observational study, participants (*n* = 226) were recruited from patients attending a dedicated BP clinic after referral from their general practitioner from Hobart, Tasmania, Australia. Referred patients typically had known or suspected hypertension and were referred for a detailed BP assessment and recommendations for management by specialist doctors. Provision of the BP values recorded by the referring doctor was a requirement and these BP values were used as the clinic BP. On attendance at the dedicated BP clinic, unobserved AOBP measurement was undertaken using an established protocol [12], and the same BP measurement device was used for 24-h ABPM at the end of the same clinic visit. Cholesterol levels were obtained from blood test reports from accredited pathology services. Additional CVD risk factors and medications were assessed via self-report questionnaire. Separate absolute CVD risk scores were calculated using the systolic BP from each measurement method (clinic BP, unobserved AOBP and ABPM). The proportion of participants classified as high risk in accordance with the 2012 Australian guidelines [1], and compared across the BP measurement methods. As a dedicated clinic for patients with suspected hypertension or suspected uncontrolled hypertension, many participants were taking anti-hypertensive medications on presentation to the BP clinic. All patients maintained their usual medication schedule between referral to the BP clinic and the clinic visit where AOBP and ABPM measures were obtained. All participants provided written informed consent and ethical approval was obtained from the Tasmania Health and Medical Human Research Ethics Committee.

### Clinic BP

Clinic BP was recorded as part of the referral process and measured in the clinic environment according to the usual practice of each referring doctor from general practice. Thus, clinic BP was not standardised and represents a real-world clinic BP measurement, instead of a standardised research-grade clinic BP as recommended to be performed by hypertension guidelines [13]. No information was available on the rest period before or between clinic BP measurements, nor the device used for clinic BP measurement. Typically, one clinic BP reading was provided, in some instances a second or third BP measurement was available. Where more than one clinic BP measurement was available the average was used for analyses.

### Unobserved AOBP

A validated automated BP device was used to obtain three unobserved AOBP measurements after 5-min of seated rest, with one-minute rest between each measurement (Mobil-O-Graph, IEM, GmbH) [12]. Participants were fitted with an appropriately sized BP cuff (based on arm circumference measurements) in a private room and advised to sit quietly with both feet on the floor, back supported and arm at heart level while the BP device automatically measured and recorded unobserved AOBP. The average of the second and third unobserved AOBP measurement was used for absolute CVD risk calculation [14].

### ABPM

The same BP device used for the unobserved AOBP protocol was also used to record ABPM (Mobil-O-Graph, IEM, GmbH) [12]. Before leaving the clinic, participants were fitted with the BP device and advised to avoid removing the cuff for the 24-h ABPM period. ABPM measurements were recorded every 20 min during the day (6am–10pm) and every 30 min at night (10pm–6am). As per guideline recommendations, participants were advised to avoid talking or moving during each BP measurement and to avoid strenuous activities during the 24-h monitoring period [15]. The average of the daytime and 24-h ABPM were used for absolute CVD risk calculation.

### White coat hypertension

Participants were classified with white coat hypertension if clinic BP was ≥140/90 mmHg and 24-h ABPM was <130/80 mmHg [16]. Sustained hypertension was classified as both clinic-BP ≥ 140/90 mmHg and 24-h ABPM ≥ 130/80 mmHg. Normotension was defined as both clinic BP and 24-h ABPM < 140/90 mmHg and <130/80 mmHg, respectively.

### Absolute CVD risk factor variables

Absolute CVD risk scores were calculated using the adjusted 5-year Framingham Risk Equation according to Australian CVD primary prevention guidelines [1, 3]. In addition to systolic BP, calculation of absolute CVD risk using the Framingham equation requires age, sex, smoking status, diabetes status, and total- and high-density-lipoprotein cholesterol.

Fasting cholesterol results were obtained from previous pathology test results on referral. For participants that did not have a recent cholesterol result (within 2 years) a sample of blood was drawn for analysis via an accredited pathology laboratory.

A health questionnaire was completed for all self-reported risk variables. Diabetes status was defined as being previously diagnosed by a medical practitioner and smoking status was defined as a current smoker or a participant who had quit smoking within the last 12 months. Height and weight were also recorded at the clinic visit.

### Absolute CVD risk calculation

Age, systolic BP, and cholesterol values were input as continuous variables. Sex, diabetes status and smoking status were entered as dichotomous variables. Four risk scores were produced for each participant with the systolic BP from each measurement method: clinic BP, unobserved AOBP, and ABPM (24-h and daytime). Participants were classified as low, moderate or high absolute CVD risk according to guideline thresholds (<10%, 10–15% and >15%, 5 year risk, respectively) [1]. In addition, participants were classified as high-risk if they reported any of the clinical criteria that denote high-risk without the need for absolute CVD risk calculation as per guideline recommendations [1]. These clinical criteria included: diabetes and aged 60 years or older, BP ≥ 180/110 mmHg, total cholesterol >7.5 mmol/L. Thresholds for hypertension for out-of-office BP measures are typically lower compared to office measures. As such, an additional analysis was conducted with AOBP and ABPM adjusted with an increase of 5 mmHg and absolute CVD risk calculation and classification undertaken as per the above steps. In addition, a lower BP threshold of ≥160/100 mmHg for AOBP and ABPM was used to classify participants at high CVD risk but the BP threshold of ≥180/110 mmHg was maintained for clinic BP to determine differences in risk classification and agreement.

### Statistical analyses

Analyses were performed in Stata version 16.1 (StataCorp, USA). Data are presented as mean (±SD) for continuous variables and as number and percentage for categorical variables. The absolute difference in absolute CVD risk scores calculated with each systolic BP were summarised and compared using the non-parametric Wilcoxon signed-rank test. Clinical significance was determined using the Cohen’s Kappa statistic (κ) to test agreement in high-risk classification according to BP, AOBP and ABPM. A κ of 0–0.20, 0.21–0.39, 0.40–0.59, 0.60–0.79, 0.80–0.90 and >0.90 was considered none, minimal, weak, moderate, strong and almost perfect level of agreement [17]. This equates to 0–4%, 5–15%, 16–35%, 36–63%, 64–81% and 82–100% of data that are reliable for none, minimal, weak, moderate, strong and almost perfect level of agreement, respectively. A sensitivity analysis was undertaken using only first clinic BP measurement for all participants compared with using the average clinic BP when more than one measurement was available. In addition, due to the relationship between advancing age and both systolic BP and absolute CVD risk a sub-analysis was undertaken to determine the effect of age on changes in CVD risk classification. *T* tests were used to compare continuous variables and Chi-squared tests were used to compare categorical variables.

## Results

### Participant characteristics

**Fig. 1:**
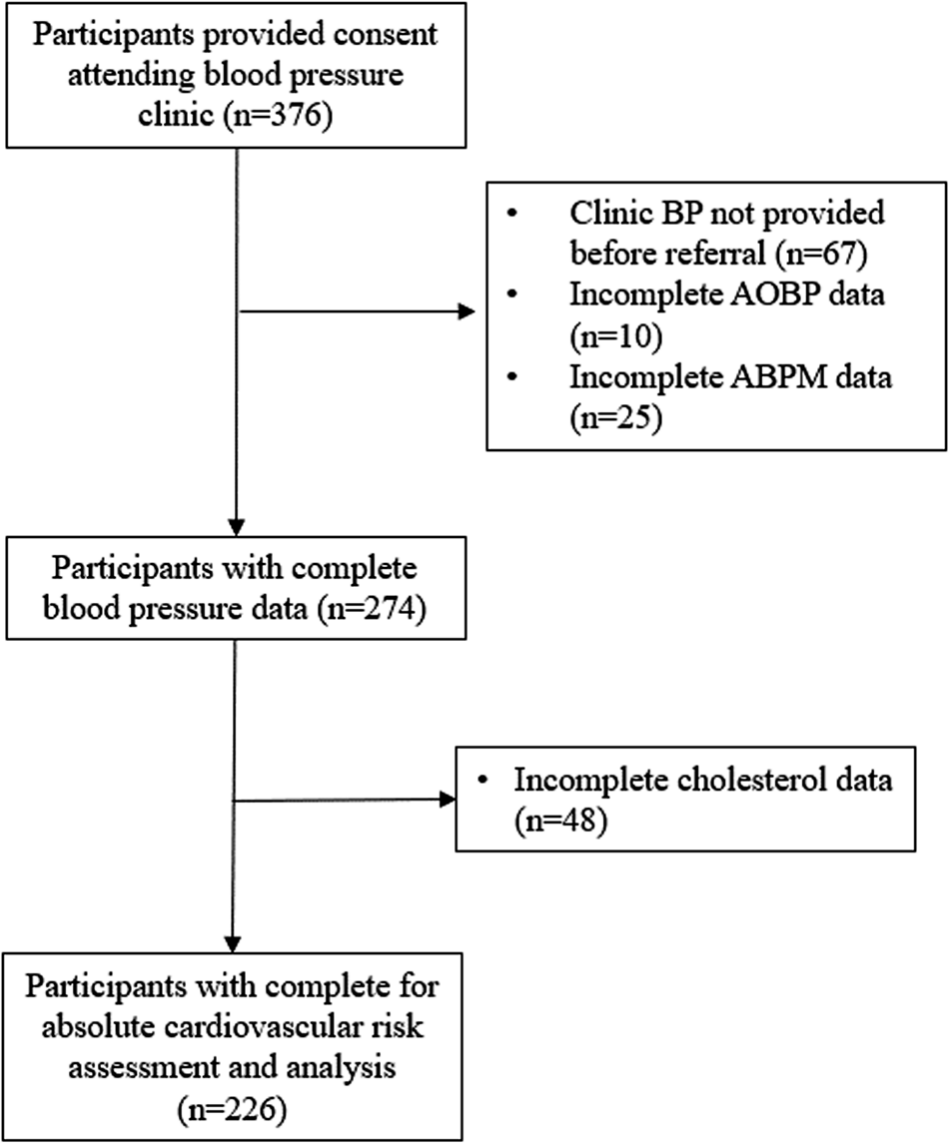
Participant flow. BP blood pressure, AOBP automated unobserved blood pressure, ABPM ambulatory blood pressure monitoring

Participant characteristics are shown in Table 1 with participant flow in Fig. 1. On average, participants were middle aged, overweight or obese and 73% were taking anti-hypertensive medications. Participants attended the BP clinic and had BP measurements taken, on average, 9 days from the time referral BP was provided. Most participants had one real-world clinic-BP (88%) provided on referral, 9% had two readings and 3% had three readings. Overall, 20% of participants were classified with white coat hypertension, 65% of those with white coat hypertension were aged 60 years or older.

**Table 1:** Clinical characteristics of patients attending a specialist blood pressure (BP) clinic where clinic BP was provided by the referring doctor and automated unobserved office BP and ambulatory BP measurement was undertaken at the first clinic visit (*n* = 226)

Variable	Mean ± SD or n (%)
Age (years)	59 ± 15
Female (% yes)	130 (58)
Body mass index (kg/m^2^)	30.6 ± 6.2
Current smoker (% yes)	25 (11)
Diabetes (% yes)	28 (12)
Total cholesterol (mmol/L)	5.2 ± 1.1
HDL cholesterol (mmol/L)	1.5 ± 0.4
**Systolic blood pressure (mmHg)**	
Clinic-BP	157 ± 19
Unobserved-AOBP	139 ± 16
24-h ABPM	135 ± 12
Day ABPM	139 ± 12
**Diastolic blood pressure (mmHg)**	
Clinic-BP	90 ± 12
Unobserved-AOBP	86 ± 11
Day ABPM	85 ± 10
24-h ABPM	82 ± 9
**Hypertension categories**	
Normotension n (%)	5 (2)
Sustained hypertension n (%)	156 (69)
White coat hypertension n (%)	46 (20)
**Medications (%)**	
Antihypertensive	164 (73)
Statins	63 (28)
**Time from referral to BP clinic assessment (days)**	9 ± 17

*SD* standard deviation, *BP* blood pressure, *AOBP* automated office blood pressure, *ABPM* ambulatory blood pressure monitoring

Differences in systolic BP and absolute CVD risk between clinic BP compared to unobserved AOBP and ABPM. Systolic clinic BP was 18 mmHg higher than unobserved AOBP and daytime ABPM and 22 mmHg higher than 24-h ABPM, respectively (Table 2). Consequently, absolute CVD risk calculated using clinic BP was >7% higher than absolute CVD risk calculated using unobserved AOBP and ABPM. Clinic systolic BP and absolute CVD risk was higher regardless of age. However, clinic systolic BP was higher among those aged ≥60 years compared to those aged.

**Table 2:** Differences in systolic blood pressure (BP) and absolute cardiovascular disease risk scores between clinic-BP, unobserved automated office (unobserved-AOBP), and ambulatory BP measurement (ABPM) (*n* = 226)

Measurement condition	Mean ± SD	Mean difference ± SD	Difference range	Z score	*P* value
**Systolic blood pressure (mmHg)**					
Clinic BP	157 ± 19	Reference			
Unobserved AOBP	139 ± 16	−18 ± 20	−43 to 71	10.76	p < 0.001
Day ABPM	139 ± 12	−18 ± 20	−48 to 75	10.42	p < 0.001
24-h ABPM	135 ± 12	−22 ± 20	−41 to 77	11.70	P < 0.001
**Absolute cardiovascular disease risk score (%)**					
Clinic BP	10.4 ± 8.1	Reference			
Unobserved AOBP	7.8 ± 6.4	−2.6 ± 3.3	−6.6 to 15.6	10.35	p < 0.001
Day ABPM	7.8 ± 6.3	−2.6 ± 3.4	−7.6 to 16.9	10.26	p < 0.001
24-h ABPM	7.3 ± 6.1	−3.1 ± 3.5	−6.4 to 17.4	11.43	P < 0.001

*SD* standard deviation, *BP* blood pressure, *AOBP* automated office blood pressure, *ABPM* ambulatory blood pressure monitoring

**Fig. 2:**
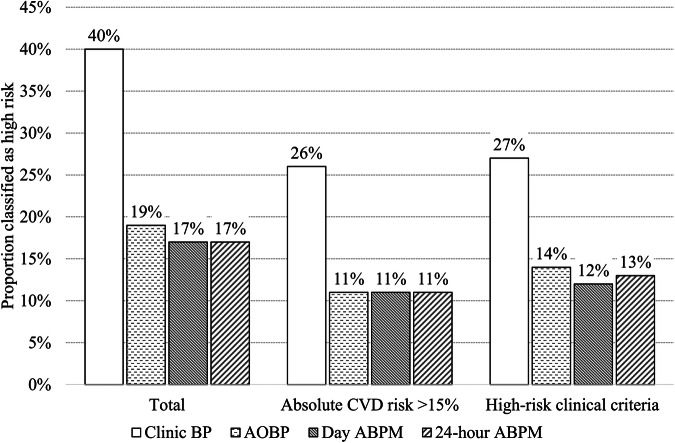
Proportion classified as high absolute cardiovascular disease risk using clinic-BP, unobserved automated office BP (AOBP) and ambulatory blood pressure measurement (ABPM) (*n* = 226)

Proportion classified as high absolute CVD risk across BP measurement methods. The proportion of participants classified at high CVD risk is presented in Fig. 2 with a full breakdown of risk classification in Supplementary Table 2. Overall, 21% and 23% more participants were classified at high CVD risk when clinic BP was used compared to unobserved AOBP and both daytime and 24-h ABPM, respectively. This increase was due to the higher clinic BP, specifically 40 participants (18%) had clinic BP ≥180/110 mmHg, of which 30 had systolic BP ≥180 mmHg and 10 had diastolic BP ≥110 mmHg. In comparison, only three participants had a systolic AOBP ≥180 mmHg and only one had a systolic daytime ABPM above this threshold. Among those participants at high risk based on clinic BP, 20% had white coat hypertension. When a lower BP threshold of ≥160/100 mmHg for AOBP and ABPM was used to classify participants at high CVD risk an additional 15% of participants were classified as high CVD risk according to AOBP and an additional 9% of participants were classified as high CVD risk according to daytime and 24-h ABPM (Supplementary Table 2). More participants aged ≥60 years were classified as high absolute CVD risk compared to those aged.

Agreement in high-risk classification according to clinic BP, unobserved AOBP, and ABPM. There was weak agreement in high CVD risk classification between clinic BP and both unobserved AOBP and ABPM (Table 3). The agreement in high CVD risk classification was strong between unobserved AOBP and ABPM (*κ* > 0.80) but moderate for absolute CVD risk calculation alone (*κ* = 0.79 [0.66–0.92]). There was no improvement in the level of agreement in risk classification when AOBP and ABPM were adjusted for absolute CVD risk classification. Among those with white coat hypertension (*n* = 46), 12 (26%) were reclassified from high CVD risk according to clinic-BP to low CVD risk when ABPM was used for absolute CVD risk calculation. The remaining 34 participants maintained the same CVD risk classification across the BP measurement conditions. The findings remained unchanged after performing a sensitivity analysis to compare high CVD risk classification using only the first clinic BP measurement for all participants and using the average clinic-BP when more than one measurement was available. In addition, agreement did not improve when a lower BP threshold (≥160/100 mmHg) was used for AOBP and ABPM to classify participants as high CVD risk (Supplementary Table 2).

**Table 3:** Agreement in high cardiovascular disease risk classification using clinic blood pressure, unobserved automated office blood pressure (AOBP), and ambulatory blood pressure measurement (ABPM) (*n* = 226)

Measurement method	Clinical criteria that denote high risk		Absolute CVD risk > 15%		Total classified as high CVD risk	
	κ (LCI-UCI)	Agreement	κ (LCI-UCI)	Agreement	κ (LCI-UCI)	Agreement
Clinic BP vs AOBP	0.57 (0.45–0.69)	Weak	0.50 (0.39–0.62)	Weak	0.50 (0.39–0.62)	Weak
Clinic BP vs day ABPM	0.52 (0.41–0.64)	Weak	0.45 (0.34–57)	Weak	0.45 (0.34–0.56)	Weak
Clinic BP vs 24-h ABPM	0.55 (0.43–0.66)	Weak	0.48 (0.37–0.59)	Weak	0.47 (0.36–0.59)	Weak
AOBP vs day ABPM	0.91 (0.78–1.03)	Perfect	0.79 (0.66–0.92)	Moderate	0.88 (0.75–1.01)	Strong
AOBP vs 24-h ABPM	0.90 (0.77–1.03)	Strong	0.79 (0.66–0.92)	Moderate	0.85 (0.72–0.98)	Strong

A κ of 0–0.20, 0.21–0.39, 0.40–0.59, 0.60–0.79, 0.80–0.90 and >0.90 was considered none, minimal, weak, moderate, strong and almost perfect level of agreement [26]. Participants were classified as high risk according to (1) clinical criteria that denote high risk without the need for absolute cardiovascular disease risk calculation; (2) a high-risk absolute cardiovascular disease risk score >15% in the total sample; and (3) an absolute cardiovascular disease risk score >15% and/or any clinical criteria that denote high risk

*AOBP* unobserved automated office blood pressure, *LCI* lower confidence interval, *UCI* upper confidence interval, *ABPM* ambulatory blood pressure monitoring, *CVD* cardiovascular disease; *κ* kappa. %

## Discussion

This is the first study in the world to examine the difference in absolute CVD risk assessment using unstandardised, real-world clinic BP compared with standardized BP protocols. The key novel finding was that the use of unstandardised, real-world clinic BP classified twice as many patients at high CVD risk when compared to absolute CVD risk assessment with more rigorous BP measurement using unobserved AOBP and ABPM. This discrepancy was due to higher systolic clinic BP, which is a common issue in clinical practice. These findings are relevant for clinical practice guidelines which currently only recommend standardised clinic BP measurements to be used for absolute CVD risk calculation. This recommendation may need to be revised if our findings are confirmed by others and supported with clinical outcome data.

There are well known challenges and several sources of measurement inaccuracy associated with real-world clinic BP measurement that result in highly variable BP readings [10, 18]. These challenges result in real-world clinic BP being vastly different from, and expected to be higher than, the highly standardised research-grade ‘clinic BP’ measurement used for the derivation of absolute CVD risk algorithms. The poor agreement between clinic BP and the standardised unobserved-AOBP and gold-standard ABPM observed in this study is unsurprising given the variability in BP measurement in clinical practice. Indeed, real world clinic BP has been shown to be 10 mmHg higher when compared to a standardised research protocol observed BP measurement or ABPM [19]. A systematic review and meta-analysis concluded that unobserved AOBP was similar to daytime ABPM and should replace traditional clinic BP measurement [20].

The benefit of unobserved AOBP is due to the removal of the white coat effect and that unobserved AOBP affords the opportunity to properly measure BP according to guideline recommended BP measurement techniques [21]. Yet, less is known about the implications of unobserved-AOBP or out-of-office measurement methods for CVD risk assessment and subsequent management. This is emerging as a critical gap in the literature as these BP measurement methods are rapidly increasing in popularity. One previous study demonstrated that 10% of patients were reclassified into a different risk category when research-grade clinic BP was used for absolute CVD risk calculation compared to home BP and ABPM [22]. An important point of difference in this current study, is the use of real-world clinic BP that often differs vastly from research setting BP measurement [23]. The twofold increase in the patients classified as high risk when using clinic BP could lead to potentially unwarranted CVD risk management strategies compared with CVD risk calculation based on higher quality BP measurement.

The need for better understanding of the role of out-of-office BP measurements is important as primary care delivery adjusts to broader adoption of telemedicine sustained after adjustment during the COVID-19 pandemic [24]. The use of telemedicine to improve BP screening and management has long been recognised with demonstrable effectiveness of BP measurement in community centres, waiting rooms, primary care settings and pharmacies [25]. Methods such as unobserved-AOBP and ABPM, facilitated through telehealth, can potentially add value to existing care pathways, improve patient risk classification and management while facilitating greater time for GPs to focus on prevention strategies. However, it is important to adapt BP thresholds as part of high-risk clinical criteria and absolute CVD risk models to reflect the difference between clinic BP and out-of-office measures as recommended by the 2017 AHA/ACC guidelines [26]. In this study, we observed a ~20 mmHg difference between in office and out-of-office BP measurements and use of a lower BP threshold for risk classification increased the proportion classified at high risk but did not improve agreement in risk classification between real-world clinic BP and out-of-office measures.

### Strengths and limitations

A strength of this study is the use of a real-world clinic BP that was provided on referral by general practitioners. This BP was measured according to the usual practice of each referring doctor. An additional strength is the use of unobserved AOBP, which has been recognised as an alternative for obtaining high-quality clinic BP measurements and that the same BP measurement device was used for unobserved AOBP and ABPM measurements. A limitation is that a standardised clinic BP measurement (according to guideline-recommended protocol over multiple visits) was not undertaken to provide a comparison of this measurement method with how BP is measured in real-world clinic practice. Other studies have shown that when clinic BP is undertaken according to a standardized protocol the BP readings are similar to unobserved AOBP and ABPM [27].

An additional limitation is that this is a small theoretical study that does not have cardiovascular event data to determine which BP measurement method is most predictive of CVD outcomes when used within absolute CVD risk calculation. Participants included those on BP lowering medications, which may have led to underestimation of absolute CVD risk. Lastly, the Framingham Risk Equation was used, but several other absolute CVD risk algorithms are available, and may have produced different results. Nonetheless, most risk algorithms use clinic BP as an input variable and most international CVD prevention guidelines include BP ≥ 180/110 mmHg as a high risk criteria without the need for absolute CVD risk assessment, so the findings are still likely to be relevant.

### Conclusion

Real-world clinic BP measurement misclassified patients at high CVD risk compared to unobserved AOBP and ABPM. Given the well-known limitations of clinic BP measurement, CVD risk management should be based on more rigorous BP measurement than clinic BP.

## Supplementary information

Supplementary Table S1

Supplementary Table 2

## Data Availability

Data is available upon reasonable request to the corresponding author (Niamh Chapman) and subject to approval from the study Principal Investigator (James Sharman).
