# Supplementary material for: Absolute cardiovascular risk assessment using ‘real world’ clinic blood pressures compared to standardized unobserved and ambulatory methods: an observational study
Source: Hypertens Res. 2024 Aug 16;47(10):2855–63. doi: 10.1038/s41440-024-01841-1 (PMC11456502; doi:10.1038/s41440-024-01841-1)
Supplement: Supplementary file 1 — Supplementary Table S1 [file 41440_2024_1841_MOESM1_ESM.docx]

Supplementary Table S1. Differences in systolic blood pressure (BP) between clinic-BP, unobserved automated office (unobserved-AOBP), and ambulatory BP measurement (ABPM) by age (n=226).

| **Measurement method** | **Participants aged <60 years (n=102)** | | | **Participants aged ≥60 years (n=124)** | | |
| --- | --- | --- | --- | --- | --- | --- |
| **Difference in systolic BP (mmHg)** | | | | | | |
|  | **Mean±SD** | **Mean difference±SD** | **Range** | **Mean±SD** | **Mean difference±SD** | **Range** |
| Clinic BP | 152±18^×^ | Reference |  | 161±19^×^ | Reference |  |
| AOBP | 138±14* | -14±18 | -24 to 69 | 141±18* | -20±21 | -43 to 71 |
| Day ABPM | 139±11* | -13±16 | -25 to 57 | 135±13* | -22±22 | -48 to 75 |
| 24-hour ABPM | 135±11* | -17±16 | -20 to 63 | 139±12* | -26±21 | -41 to 77 |
| **Difference in absolute CVD risk (%)** | | | | | | |
|  | **Mean±SD** | **Mean difference±SD** | **Range** | **Mean±SD** | **Mean difference±SD** | **Range** |
| Clinic BP | 5.7±5.5^×^ | Reference |  | 14.2±7.9^×^ |  |  |
| AOBP | 4.4±4.5*^×^ | 1.3±1.9 | -1.4 to 11.5 | 10.6±6.4*^×^ | 3.6±3.8 | -6.6 to 15.6 |
| Day ABPM | 4.6±4.5*^×^ | 1.2±1.9 | -3.4 to 10.9 | 10.4±6.4*^×^ | 3.8±3.9 | -7.6 to 16.9 |
| 24-hour ABPM | 4.2±4.2*^×^ | 1.5±2.1 | -2.7 to 13.0 | 9.8±6.2*^×^ | 4.4±3.9 | -6.4 to 17.4 |
| **High cardiovascular disease risk classification n (%)** | | | | | | |
|  | **Clinical criteria** | **Score** | **Total** | **Clinical criteria** | **Score** | **Total** |
| Clinic BP | 18 (18) | 5 (5) | 23 (23) | 42 (34) | 24 (19) | 66 (53) |
| AOBP | 4 (4) | 3 (3) | 7 (7) | 28 (23) | 9 (7) | 37 (30) |
| Day ABPM | 3 (3) | 4 (4) | 7 (7) | 26 (21) | 5 (4) | 31 (25) |
| 24-hour ABPM | 3 (3) | 4 (4) | 7 (7) | 24 (19) | 7 (6) | 31 (25) |
| Abbreviations: SD, standard deviation; BP, blood pressure; AOBP, automated office BP; ABPM, ambulatory BP monitoring.  * indicates a statistically significant difference (P<0.001) between blood pressure measurement categories within a single age category.  ^×^ indicates a statistically significant difference (P<0.05) between age categories for the same variable. | | | | | | |
