# Supplementary material for: Absolute cardiovascular risk assessment using ‘real world’ clinic blood pressures compared to standardized unobserved and ambulatory methods: an observational study
Source: Hypertens Res. 2024 Aug 16;47(10):2855–63. doi: 10.1038/s41440-024-01841-1 (PMC11456502; doi:10.1038/s41440-024-01841-1)
Supplement: Supplementary file 2 — Supplementary Table 2 [file 41440_2024_1841_MOESM2_ESM.docx]

Supplementary Table 2. Proportion classified as high absolute cardiovascular disease risk using clinic-BP, unadjusted and adjusted unobserved automated office BP (AOBP) and ambulatory blood pressure measurement (ABPM) (n=226).

| **Variable** | **Clinic-BP n (%)** | **AOBP n (%)** | **Day ABPM n (%)** | **24-hour ABPM n (%)** |
| --- | --- | --- | --- | --- |
| **Classification of CVD risk using unadjusted AOBP and ABPM readings** | | | | |
| 1. **Classified as high-risk according to clinical criteria that denote high risk:** | | | | |
| Diabetes and aged ≥60 years | 21 (9) | 21 (9) | 21 (9) | 21 (9) |
| Total cholesterol ≥7.5 mmol/L | 6 (3) | 6 (3) | 6 (3) | 6 (3) |
| BP ≥180/110 mmHg | 40 (18) | 5 (2) | 2 (1) | 0 |
| Total | 60 (27) | 32 (14) | 29 (13) | 27 (12) |
| 1. **Classified as high risk according to absolute CVD risk score (>15%):** | | | | |
| Total sample | 58 (26) | 25 (11) | 24 (11) | 24 (11) |
| Without high-risk clinical criteria | 29 (13) | 12 (5) | 9 (4) | 11 (5) |
| **Total classified as high CVD risk** | 89 (40) | 44 (19) | 38 (17) | 38 (17) |
| **Classification of CVD risk using adjusted AOBP and ABPM readings** | | | | |
| 1. **Classified as high-risk according to clinical criteria that denote high risk:** | | | | |
| Diabetes and aged ≥60 years | 21 (9) | 21 (9) | 21 (9) | 21 (9) |
| Total cholesterol ≥7.5 mmol/L | 6 (3) | 6 (3) | 6 (3) | 6 (3) |
| BP ≥180/110 mmHg | 40 (18) | 6 (3) | 2 (1) | 1 (0.5) |
| Total | 60 (27) | 32 (14) | 29 (13) | 28 (12) |
| 1. **Classified as high risk according to absolute CVD risk score (>15%):** | | | | |
| Total sample | 58 (26) | 33 (15) | 29 (13) | 26 (12) |
| Without high-risk clinical criteria | 29 (13) | 16 (7) | 13 (6) | 12 (5) |
| **Total classified as high CVD risk** | 89 (40) | 48 (21) | 42 (19) | 40 (18) |
| 1. **Agreement in total classified as high CVD risk** | | | | |
| *κ (LCI-UCI)* | REF | 0.55 (0.43 to 0.67) | 0.48 (0.36 to 0.59) | 0.48 (0.36 to 0.59) |
| *Agreement level* | REF | Weak | Weak | Weak |
| **Classification of CVD risk using a lower BP threshold of ≥160/100 mmHg for AOBP and ABPM** | | | | |
| BP ≥160/100 mmHg | REF | 35 (15) | 21 (9) | 10 (4) |
| Total classified as high CVD risk | REF | 66 (29) | 54 (24) | 47 (21) |
| Agreement *κ (LCI-UCI)* | REF | 0.43 (0.30 – 0.55) | 0.47 (0.35 – 0.59) | 0.45 (0.34 – 0.57) |
| Agreement level | REF | Weak | Weak | Weak |
| Abbreviations: AOBP, automated unobserved blood pressure, ABPM, ambulatory blood pressure monitoring, CVD, cardiovascular disease.  AOBP and ABPM were adjusted by adding 5mmHg to all measurements as a correction for different BP measurement methods. | | | | |
